# The role of the assistant practitioner in the clinical setting: a focus group study

**DOI:** 10.1186/s12913-018-3506-y

**Published:** 2018-09-10

**Authors:** Catherine Henshall, Andrea Doherty, Helen Green, Liz Westcott, Helen Aveyard

**Affiliations:** 1 OxINMAHR, Faculty of Health & Life Sciences, Oxford Brookes University, Oxford, OX3 0BP UK; 2 Learning and Education, Manor House, John Radcliffe Hospital, Oxford University Hospitals NHS Foundation Trust, Headley Way, Oxford, OX3 9DU UK; 3 Oxford Health NHS Foundation Trust, Unipart House, Garsington Road, Oxford, OX4 2PG UK; 4 Faculty of Health & Life Sciences, Oxford Brookes University, Oxford, OX3 0BP UK

**Keywords:** Assistant practitioner, Registered nurse, Role clarity, Role identity, Nursing associate, Career development, Career pathways, Staff structure

## Abstract

**Background:**

Assistant practitioners have knowledge and skills beyond the level of traditional support workers, and work in many clinical settings. However, some assistant practitioners lack a clearly defined role and may be under-used due to issues around accountability and uncertainty about their purpose. This paper explores the assistant practitioner role from the perspectives of assistant practitioners and registered nurses.

**Methods:**

This study aimed to explore the role of the assistant practitioner from the perspectives of assistant practitioners and registered nurses in two NHS hospital trusts in Oxfordshire, United Kingdom. Six qualitative focus groups were undertaken between February–March 2017. Ethical approval was obtained (FREC 2016/05) and written consent was provided by participants. Data was analysed thematically analysed using the Framework method.

**Results:**

Nineteen participants (assistant practitioners, *n* = 12; registered nurses, *n* = 7) were recruited using convenience sampling. Emerging themes related to ‘fluctuating roles and responsibilities of assistant practitioners’, ‘role differences between registered nurses and assistant practitioners’, ‘working relationships’, ‘supervision’ and ‘redefining nursing pathways’.

The Results and Discussion sections highlight a lack of role clarity and blurring of boundaries between the roles of assistant practitioners and registered nurses, with many tasks undertaken by both. This lack of ownership of ‘nurse-specific’ roles by registered nurses was evident and clear differences were only encountered with regard to accountability. The development of the Nursing Associate role provides managers with the opportunity to redefine staff banding hierarchies to ensure that clinical staff are aware of their role capabilities and limitations and are practicing safely, whilst promoting career development and progression pathways.

**Conclusion:**

Addressing issues around role clarity can benefit professional development, satisfaction, role identity and ownership for registered nurses and assistant practitioners, by recognising the individual and collective value they bring to the clinical team. The findings can help inform the development of the Nursing Associate role.

## Background

Assistant practitioners (APs) are higher level support workers who complement the work of registered health professionals [1]. APs have knowledge and skills beyond the level of traditional support workers, such as health care assistants (HCAs), and deliver some aspects of care which were formerly the domain of registered staff. Working under the supervision of registered health professionals, AP roles in the health and social care sector include direct patient care, day to day patient management and broader aspects of healthcare, such as involvement in health promotion [2].

APs are most commonly employed at a Band 4 level following education and training, usually via a Foundation Degree or an equivalent programme, which is undertaken in partnership with local Higher Education Institutes [3]. This educational base creates a platform for greater knowledge, opportunity and capability in the clinical setting, in comparison to HCAs, who do not undergo this formal training programme. The AP role is cross-disciplinary, rather than nursing specific [4] with APs working in a variety of settings including general practice [5], critical care [6] perioperative practice [7, 8] radiography [9] and social care [10]. Whilst the role has the potential for adoption in many settings, evidence suggests that APs are most prevalent in areas where funding is available for them, rather than in areas where a need for them has been identified [11].

As band 4 s, APs are currently non-registered healthcare staff and are not subject to the professional regulation of registered health professionals. However, by working to locally agreed and defined protocols APs do possess a certain level of accountability to themselves, their employers and the people they care for [12, 13].

In their study of the work of APs, Wakefield et al. (2010) identified that their role usually falls into one of three categories - supportive/assistive to the registered practitioner, substitute to the registered practitioner and/or acting autonomously [14]. The study indicates that some APs are working without appropriate supervision and often lack a clearly defined role. This lack of role clarity, alongside blurred role boundaries between APs and HCAs and APs and registered nurses (RNs) has been well documented [3, 15–18]. Studies have also found a lack of consistent terminology when describing the AP role, with the terms ‘assistant’ and ‘associate’ practitioner often used inter-changeably [16]. Similarly, Miller et al. (2015) found 15 different terms to describe the role [11].

Possibly due to their lack of role clarity Thurgate et al. (2013) found that APs interpreted and created their own clinical roles [19]. Furthermore, Miller et al. (2014) identified that in practice it was difficult to distinguish between the work undertaken by bands 2–4 and cited this as a possible cause of workforce friction [20]. Despite, some APs reporting ‘widened horizons’ upon commencing in their new roles, friction between APs and other members of the healthcare team have been widely reported [21]. In addition, whilst APs often accept a subordinate role and defer to nursing staff, it has been suggested that the undertaking of duties that were once considered the exclusive role of the RN is seen by some APs as a position of privilege, without the administrative burdens faced by RNs [22].

Spilsbury et al. (2011) evaluated the development and impact of APs supporting ward-based RNs in the National Health Service (NHS) [23]. The research consisted of case studies at three acute NHS Trusts, using quantitative and qualitative methods, including focus groups and interviews, followed by a national survey of APs [23]. It concluded that APs made a valuable contribution to patient care but that their role was dependent on the ability of RNs to recognise their potential. In addition, the role of the AP and the tasks undertaken by them were heavily influenced by the number of RNs on duty; a greater number of RNs led to a restriction in AP roles and responsibilities [23]. The study also suggested that APs are underused because RNS are uncertain about issues relating to accountability and because of uncertainty about the purpose of the AP role throughout the wider team [23].

## Aim of the study

This study has aimed to explore the role of the AP from the perspective of both APs and RNs working in acute, community and mental health settings in two NHS hospital trusts in Oxfordshire, United Kingdom. It will contribute to the growing body of literature around the AP role and how it co-exists alongside that of the RN. This will enable discussion around what the role does, and should, look like both now and in the future, which is particularly pertinent in light of the emerging Nursing Associate (NA) role. The findings from the study will have the potential to inform the development, credibility and successful implementation of this new healthcare initiative.

## Methods

### Study design

A qualitative focus group study design was chosen to explore the role and scope of the AP in the clinical setting from the perspectives of both APs and RNs. Focus groups were chosen, to allow participants the opportunity to explore their own and others’ perspectives collectively, with opportunity for shared discussion and debate. In addition, focus groups allowed the researchers to gather data from a variety of APs and RNs working across the two participating hospital Trusts within a relatively short time frame, something which would have been more difficult with one-to-one interviews, due to time pressures on healthcare staff. Six focus groups were conducted in total. Of these, three were carried out in each participating Trust; two for APs and one for RNs. Separate focus groups were undertaken for APs and RNs to allow the participants to speak freely without feeling inhibited in the presence of other group members, thus facilitating discussion.

### Setting

The study was commissioned by the two participating NHS Trusts and took place between February and March 2017. It was undertaken as a means of evaluating the introduction of the AP role across the two Trusts. Ethical approval was sought and obtained from Oxford Brookes University Research Ethics Committee in October 2016 (FREC 2016/05). HRA approvals were obtained in December 2016 and permissions from the two local Research and Development departments were subsequently obtained in January 2017 (IRAS number 214408). Individual written consent was taken from participants before they undertook the focus group study.

### Access, recruitment and sampling

APs and RNs working in the two Trusts were invited to participate in the focus groups. Convenience sampling was used to recruit participants to the study. Participants in community, mental health and acute settings were accessed through the researchers contacting AP line managers to seek their permission for their staff to be approached to participate. Once permission was granted line managers were provided with copies of the participant information leaflets via email, which detailed the study purpose and were asked to distribute it to any members of staff who were eligible to participate. Potential participants were asked to contact the researchers if they were interested in participating in the study. Following this, times and locations for the focus groups were scheduled that were convenient for the majority of participants who were hoping to attend. Focus groups were held on Trust premises across the region to accommodate the preferences and travel constraints of most participants.

The focus groups were held in meeting rooms at the two participating hospital Trusts and refreshments were provided. Each focus group lasted around one hour and was informed by a topic guide devised by the researchers that covered issues relating to the role and scope of the AP in the clinical setting (Table 1). Each focus group was moderated and facilitated by two researchers from the team.

**Table 1:** Examples of questions from topic guide used to undertake focus groups with APs and RNs

• Can you describe the clinical settings you work in at the Trust? • Can you tell me what your role as APs/RNs involves on a day to day basis? • If you are an AP, were you the first in your area? How was the role introduced; what could be improved? • What are the core roles and responsibilities APs should possess regardless of the clinical setting? • What skills do you feel are setting specific? How should these be decided on as AP rather than RN skills? • How closely do you work alongside APs/RNs on a day to day basis? • Can you give me some examples of roles that are distinctly AP roles, rather than RNs and vice-versa? • How does the AP role differ from that of other unregistered HCAs? • How clear is the role task allocation between APs and RNs? How is this decided? Has it changed? • Has the RN role changed since APs started working clinically? How has this affected clinical practice? • If you are an RN, has the introduction of the AP role changed how you view your own role? • What level of support/clinical supervision do you feel you receive from RNs/APs? • How much autonomy do you feel you have in your role as an AP? • How accountable do you feel for your own practice as an AP/the practice of APs as an RN? • Do you feel there are opportunities for career development/progression as an AP? If so, in what way? • What impact do you feel the AP role has on clinical care outcomes in terms of quality of care, patient safety, service efficiency and effectiveness, organisational pressures and best practice?	

### Data analysis

The focus groups were recorded using a digital voice recorder before being transcribed by a local transcription service. All identifying participant details were anonymised during the transcription process and the focus group recordings were removed from the voice recorder once they had been uploaded for transcription. Copies of the digital recordings were stored on the university computers in line with local data protection policies. Data was analysed thematically and managed using the Framework method, with its matrix output providing a structured and systematic way for managing and analysing the qualitative data [24]. Transcripts were double-coded by two members of the research team (CH, HA). Following this a working analytical framework was established and elements of the constant comparative method were used, with the researchers iteratively moving back and forth between data collection and analysis [25]. This enabled the researchers to establish any similarities and differences in perspectives between APs and RNs, in relation to the role and scope of the AP in clinical practice. Transcript data were inserted into a Framework matrix to enable ordering and data synthesis of the data [24] and this enabled within and across case analysis of the data from the six focus groups. Once both researchers had analysed the dataset, they met to compare their findings and to talk through any emerging themes arising from the participants’ discussions. Following this, a further meeting was held with all members of the research team to discuss, explore and develop these emerging themes further. Through studying the focus group data in this level of detail, we were able to draw out relevant themes relating to participants’ views on the role and scope of the AP in the clinical setting. The findings will now be reported on.

## Results

### Summary of main themes

Nineteen participants (APs, *n* = 12; RN, *n* = 7) attended the focus groups. A summary of participant characteristics is provided in Table 2. APs and RNs worked in a wide and diverse range of acute, community and mental health settings across the two Trusts including forensics, dialysis, acute vascular, endoscopy, breast, ambulatory care, gynaecology, cardiothoracic, orthopaedic and emergency assessment and district nursing.

**Table 2:** Characteristics of Focus Group Participants

Participant Characteristics		Participants (*n* = 19)
Clinical Role	Assistant Practitioner	12
	Registered Nurse	7
Gender	Female	18
	Male	1
Age (years) ^a^	20–29	0
	30-39	1
	40–49	8
	> 50	4
NHS Trust	Acute	11
	Community and Mental Health	8
Band	4	12
	5	2
	6	1
	7	3
	8	1

^a^Data for age of participants incomplete

The main themes to emerge from the dataset related to the fluctuating roles and responsibilities of the APs, key differences in the roles of RNs and APs, working relationships between RNs and APs, supervision of APs and redefining nursing pathways.

### Fluctuations in AP roles and responsibilities

Participants revealed that the roles of APs, both across and within the two hospital Trusts, were multifaceted and covered a wide range of clinical tasks and responsibilities. These included, but were not limited to, undertaking ECGs, venepuncture, cannulation, pressure area care, wound dressings, administering injections and assisting with activities of daily living. In addition, some APs also took on extended roles which included admitting and assessing patients, overseeing bed management duties, making patient referrals, assisting with paperwork and providing specialist clinical advice to patients, for example through the provision of anticoagulation advice.

However, APs had varying levels of clinical responsibility that were not always consistent with that of peers in nearby units or departments. For example, in some clinical areas, such as the community, APs administered medications intravenously, catheterized patients or provided teaching sessions to HCAs, whereas in other areas this was deemed beyond their competency boundaries and requirements. One AP commented:

*‘The role can be as diverse as the setting that you’re working in, depending on who you’re working with; how far they want you to... they want to take you with your competencies.’ (AP focus group 1).*

Some APs described how some clinical roles such as skin care assessments, teaching roles, administering flu vaccinations and caring for sedated patients, that they had previously undertaken and gained competencies in, had been revoked, due to their current line manager deciding the roles were beyond the remit of a band 4 practitioner. Some RNs expressed their concern at this ‘de-skilling’ of the AP workforce, feeling it had the potential to leave APs feeling undervalued.

*‘I think it’s important that they feel valid and then they don’t have skills taken away from them.’ (RN focus group 2*).

However both RNs and APs commented that when RN staffing levels were low, APs were often counted back in the numbers and expected to take on these once relinquished roles and responsibilities. Participants commented that it contributed to AP role ambiguity, as other nursing colleagues couldn’t keep up with what APs could and couldn’t do. This selective use of their expertise led to participants feeling that APs were sometimes used and taken advantage of.

*Participant 1: ‘If we’re short then they’ll be put in as a role…One day it’s good enough for them to do something…And then the next day it’s...When we’ve got enough, when we’ve got another trained on, oh, you can go back to doing that then. I mean, it’s just a bit...’*

*Participant 2: ‘Just to suit needs really.’ (RN focus group 3).*

Most RNs felt that the inconsistency surrounding which roles APs were allowed to undertake within and across the hospital Trusts was due to local decision-making, often dependent on what individual managers felt to be within the scope of a band 4 role rather than national role descriptions. In addition, these RNs felt that many AP competencies had been designed to be setting specific, leading to the huge diversity between the skill sets of APs across different clinical areas. It was felt that this could make it more difficult for APs to transfer to other clinical areas. Both APs and RNs described a need for increased role clarity for APs at an organisational level, through the development of a range of core competencies, to increase the transferability of AP skills.

*‘Doing the competency work right from the beginning and Trust wide I think is helpful rather than it being, kind of, each unit or each service…Then you end up with it being very different…The idea is that we’d have sort of core competencies and then there would be some specifics for the area. But they would be understood at a ward level and a service level and a Trust level which I think will hold much more power.’ (RN focus group 1).*

### Key differences between the AP and RN roles

Participants identified some key differences between RN and AP roles, the main one being that APs could not dispense medication, as they did not hold NMC registration. Yet, some RNs and APs suggested that there were few differences between their roles, apart from medication administration and discharging patients.

*‘The nurses do the same as me. They can discharge the patients, though, from the ward and also they administer the drugs and look after the controlled drugs. But, other than that, most things I can do’. (AP focus group 2).*

However, other responsibilities were identified that were considered to be largely, though not always, the domain of the RN. These included administering intravenous saline, performing palliative care, undertaking initial assessments, first visits, discharges, dispensing controlled drugs and administering certain injections, such as insulin. RNs were also identified as being the named nurse for their patients due to their professional registration and accountability. Whilst for most APs this came as a relief, RNs described feeling accountable for the actions of APs as their registration depended on them ensuring that APs worked in a professional and competent manner, despite the frequent overlap in their clinical roles and responsibilities. One RN commented:

*‘Everything is so blurry and then at the end of it, it’s still accountable to the nurse.’ (RN focus group 3).*

The blurring of boundaries between AP and RN roles had at times caused tensions, with one AP commenting that some RNs she had worked with had been unable to accept that she was able to take on some aspects of the RNs role, resulting in clarification at managerial level being sought. Other APs gave examples of doctors assuming that APs could dispense medications and of uncertainty and grey areas around what distinguished APs from both RNs and HCAs.

*‘A little while back I had some issues in my department. Nurses not seeing that I could actually do certain parts of the role so we had to have some sort of clarification for what I can and can’t do so that all the staff know what they can ask me to do and what I can’t do.’ (AP focus group 2).*

### Working relationships between APs and RNs

The majority of RNs spoke positively about how APs had integrated in to the clinical team, describing how they ‘get on with it’ are ‘highly skilled’ and ‘fit into the team well.’ APs also described strong working relationships with RNs and spoke of how they helped RNs to offload some of their clinical tasks that HCAs were unable to take on, such as cannulation, so RNs had more time to spend on other aspects of patient care. In this way RNs and APs felt they went some way to bridging the gap between RNs and HCAs.

*‘APs just get on with it and fit in well with the team. And even though they may not be doing medication…They actually do have a list of what they need to do and they link in well with the nurses and say like delegating and kind of picking up things that aren’t done.’ (RN focus group 2).*

The level of integration between APs and RNs was described as particularly high in the community, attributed to the planning and infrastructure that had been implemented by community leads to support their APs. Initiatives such as regular AP updates at band 6 meetings, regular group and individual AP supervision and AP competency planning were all viewed as key in helping APs to integrate fully into their community roles alongside RNs. Although APs and RNs acknowledged that RNs worked more autonomously than APs, some RNs commented that many APs were more experienced and used more initiative than some of their fellow nursing colleagues.

*‘Both of the APs that we’ve got do take on more in the department and maybe I shouldn’t be saying it but sometimes they take on a bit more than some of our qualified nurses.’ (RN focus group 3).*

Some APs felt that although they had developed good working relationships with RNs over time, they had experienced initial distrust and suspicion from RNs who felt that APs were trying to take over their roles. APs spoke of having to ‘prove themselves’ to RNs over time before any responsibility was relinquished to them. Though in most cases this had resulted in positive working relationships, a few APs spoke of how they felt some of their RN colleagues were unable to differentiate between the role of the AP and the HCA and were unable to comprehend what their role entailed.

*‘It’s not just: hey presto, you’re autonomous, you know, you build up your... your colleagues get to know you... You have to prove yourself.’ (AP focus group 1).*

### Working under supervision

Most participants acknowledged that APs made decisions in conjunction with, rather than independently of RNs. However, the level of independent working varied from role to role. Some APs described working very closely with RNs on a daily basis, whilst others described spending much of their time doing home visits independently as lone workers in the community. This led to infrequent supervision and as a result, APs working in this context often made clinical decisions. One AP spoke of how she worked semi-autonomously, with oversight but not direct supervision from, RN.

*‘On a day to day basis, not very often we work alongside anybody, other than, when you discuss something in handover. I suppose that’s the only time when you talk about something you’ve encountered, or you talk about patients, that would be the only time.’ (AP focus group 1).*

This often remote and relatively infrequent supervision from RNs meant that often APs were called to use their judgement to make decisions about patient care. One AP commented: ‘

*‘The unit is very busy and although I have the support there, sometimes I have to use my skills to make the situation appropriate. But I can do things like using my initiative.’ (AP focus group 2).*

### Redefining nursing pathways

Some RNs commented that understanding of the AP role across the two Trusts was limited as there had never been any formal acknowledgement as to what the role should look like at the outset. A few APs described feeling unsupported when they started in new roles due to none of their colleagues knowing what the purpose of an AP was, leading to ambiguity. Another AP spoke of how her colleagues didn’t know what to do with her when she started in her new role.

*‘They didn’t really know what to do with me when I first came into the district nursing team. There wasn’t a specific role for me. So, it was quite confusing.’ (AP focus group 1).*

RN participants described a need to clearly define competencies for all clinical staff bandings, as a means of clearly demarcating their different role expectations. They spoke of how the current banding system was unclear and ambiguous and had been detrimental to the development of the AP role when it was initially established. One RN commented that it was difficult to see how the AP role fitted into the existing nursing structure, largely as it was never budgeted for or considered in the staffing numbers when the role was developed, leading to APs being slotted in to vacant posts in an ad hoc and unstructured way. Some of the RNs were keen to learn from the unstructured and inconsistent way the AP role had been developed, by delineating clearly from the outset what the upcoming NA role should consist of, to enhance role clarity and purpose.

*‘I think that there will be lessons that we’ve learned then that now we’ll need to do this properly…Because one you’ve got to develop it but then you’ve also got …To get people to understand what [the Nursing Associate role] is...And I don’t think you can do that without kind of reviewing all your roles and structures as to how they fit in. And... Just people understanding what that role is.’ (RN focus group 1).*

This lack of role identity had led to some APs feeling that they had to fight to ‘prove their worth’ and demonstrate their level of expertise in their relevant clinical areas. This had added to feelings of uncertainty for some APs in terms of job security and career progression, with some expressing a lack of career development opportunities since becoming APs. As a result, some APs commented that they would consider undertaking the newly developed NA training, despite feeling it was very similar to their own AP training, as they felt that the NA role would be seen as more high profile. In addition, some were concerned that the NA role would result in a phasing out of the AP role, as it was regulated by the NMC, whereas the AP role was not. APs spoke of the importance of considering the differences between their role and that of NAs, as they were both band 4 roles within the discipline of nursing. Some also felt it was wrong to have the word ‘nurse’ in the NA title as it would lead to confusion about who is and is not qualified.

*‘Having an [Nursing] Associate role …Does that…Make it difficult as to what is the NA role and what is the AP role and what is the difference? They are all the same banding, the same discipline and what’s the difference?’ (RN focus group 1).*

Some APs reported feeling stuck as they didn’t know how to progress further in their roles. They described a sense of uncertainty as to whether the onus was on themselves or their managers to take responsibility for their role development, with the pro-active nature of individual APs recognised as a marker of successful development. Other APs and RNs spoke of developmental opportunities such as undertaking nurse training or apprenticeship pathways as vehicles for progression, as well as moving into non-clinical band 5 jobs such as administrative, finance and discharge planning roles.

*‘They actually want people to be employed as apprentices, and then, move up the ladder that way…They feel that there’s going to be more bang... more opportunities for band 4s within the trust, within the NHS…Because, that’s what the government wants.’ (AP focus group 1).*

## Discussion

Our findings indicate the existence of largely collaborative, positive and flexible working partnerships between RNs and APs. However the findings have also highlighted a continuing and substantial lack of role clarity faced by APs in the clinical setting [14, 23]. Many RNs and APs did not feel there were many differences between their roles and were only able to articulate a handful of distinguishing features. APs and RNs made numerous references to a blurring of boundaries between the roles and whilst some tasks were identified as discrete roles for RNs, the majority were deemed suitable for both APs and RNs. This lack of ownership of ‘nurse-specific’ roles by RNs was evident and clear; differences were only encountered with regard to accountability. This suggests that whilst RNs may be more accountable than APs, in terms of delegating clinical roles and being responsible for overall patient care, their day to day clinical tasks may not differ greatly.

Lack of clarity between the roles of registered and unregistered staff has been well documented in the nursing literature with the acknowledgement that RNs regularly undertake duties that could be performed by unregistered staff, who in return often undertake ‘nursing’ duties [26–30]. Whilst it is widely acknowledged that the RN’s role is becoming increasingly diverse and difficult to neatly define [31–33], attempts must be made to delineate what the core credentials of being a nurse really are. Studies have shown that role ambiguity results in poor performance, stress and problems in the retention of staff [26–28, 34–36]. With increasing pressure on healthcare services, it is imperative that RNs and APs are supported in their work through clearly demarcated roles which will enable them to take professional ownership and develop within their own clinical parameters.

The findings revealed that an apparent lack of AP role definition and clarity had been evident from the start of the AP training programme, with APs being slotted into vacancies as they arose rather than identified as a vital component of healthcare teams. The recent launch of the band 4 NA role, which has been introduced to bridge the gap between HCAs and RNs is similar to the AP role, with one key difference being that NAs, unlike APs, will be Nursing and Midwifery Council regulated [37, 38]. Whilst some APs expressed concern that the NA role might result in the phasing out of the AP role, RNs were keen to ensure that steps were taken and lessons learned to provide NAs with clear developmental pathways. This will ensure that NAs are secure in the responsibility, accountability and relevance of their roles from the outset. The development of the NA role provides managers at an organisational level the opportunity to unpick, reshape and reform the banding hierarchies of clinical staff, so that these boundaries become less blurred and more defined. This blurring has been illustrated in the findings by the multitude and ever-changing roles APs described across their different clinical settings. Whilst it is essential that registered and unregistered staff work together at all levels, distinctions between bands are imperative to make sure that clinical staff are aware of their role capabilities and limitations and are practicing safely, as well as to promote clearly defined pathways for career development and progression.

Another important consideration to emerge from the findings relates to the varying levels of supervision APs received from RNs, with supervision ranging from working closely alongside RNs on a day to day level, to checking in with a nurse just a couple of times a week. Whilst autonomous working practices can be advocated on many levels, the findings have highlighted the increasing clinical roles and responsibilities that are being undertaken by APs. Therefore caution must be exercised to demonstrate and ensure that APs are working within and not beyond their competencies and skill sets. Even where competencies have been met, close oversight and guidance from RNs is imperative to ensure that APs are fully supported and are provided with opportunity to develop and learn. Whilst supervision practices will vary between clinical settings, a lack of time or conflicting clinical priorities are not sufficient reasons to minimise or dismiss their importance.

The up-skilling of APs when the clinical areas they are working in are short staffed, to enable them to undertake tasks that are primarily reserved for the RN, further highlights the apparent lack of role clarity between RNs and APs. Whilst this up-skilling could be argued as a way of making the best of under resourced staffing issues, it does nothing to validate either RNs or APs, discrediting the specialist skills of RNs whilst capitalising on the good will of the AP workforce. This ‘stepping up’ displayed by APs is reminiscent of the old Senior Enrolled Nurses (SENs) role, whereby SENs were only allocated responsibility for the ward in the absence of the RN [39].

With the advent of degree level qualifications only recently deemed a necessity for all UK trained nurses (Royal College of Nursing, 2017), the up-skilling of APs and introduction of NAs into the clinical arena creates another route into nursing for staff who wish to pursue an apprentice style model. However, by investing in the skill sets of band 4 nursing staff, there is a possibility that the prevalence of band 5 nurses, who are already under-resourced, may become scarcer still, with qualified nursing jobs being predominantly reserved for band 6 nurses or above. Whilst this may make financial sense to the NHS in the short term, it is important that graduate nurses see a clear career route and a pathway for progression on entering the nursing register, if recruitment and retention rates are to be sustained. The proliferation of APs and NAs throughout the health service could be seen as an inherent threat to RNs, devaluing the skills they possess as commonplace and easy to obtain. Whilst the education, knowledge and skills of APs and NAs should not be undervalued, care must be taken to ensure that the different roles of APs, NAs and RNs are considered, clarified and developed fully to allow each of these essential roles to be harnessed and nurtured to their full potential and to maximise patient care outcomes.

### Strengths and limitations

This study sampled RNs and APs from across a wide variety of clinical settings, including acute, mental health and community trusts and therefore the findings can be applied to a range of clinical nursing areas. In addition the study gained the perspectives of both APs and RNs, which enabled the researchers to identify similarities and differences in the views and perspectives of RNs and APs; this strengthens the qualitative methods undertaken. However, due to the large geographical area the two NHS trusts spanned, we were unable to recruit as many participants to the study as planned, largely due to difficulties in participants travelling to the focus group locations due to time pressures. However, despite this the focus groups were able to generate and uncover a breadth and depth of rich experiences relating to this important topic.

## Conclusions

This paper has reported on a study exploring the role and scope of the AP in the clinical setting from the perspectives of APs and RNs. The overarching finding from the study is the crucial need for role clarity to be embedded within the role of APs, RNs and the new NA role. In addressing this on-going problem, benefits can abound in terms of professional development, satisfaction, role identity and ownership and excellence in patient care. Similarly, for the wider profession of nursing, clearly delineated roles and responsibilities between staff bandings has the potential to substantiate nursing on all levels and validate the role of APs, NAs and RNs, by recognising the individual and collective value they bring to the clinical team.

## Abbreviations

AP: Assistant practitioner
NA: Nursing associate
NHS: National Health Service
RN: Registered nurse
